# UK consensus on pre-clinical vascular cognitive impairment functional outcomes assessment: Questionnaire and workshop proceedings

**DOI:** 10.1177/0271678X20910552

**Published:** 2020-03-09

**Authors:** Aisling McFall, Tuuli M Hietamies, Ashton Bernard, Margaux Aimable, Stuart M Allan, Philip M Bath, Gaia Brezzo, Roxana O Carare, Hilary V Carswell, Andrew N Clarkson, Gillian Currie, Tracy D Farr, Jill H Fowler, Mark Good, Atticus H Hainsworth, Catherine Hall, Karen Horsburgh, Rajesh Kalaria, Patrick Kehoe, Catherine Lawrence, Malcolm Macleod, Barry W McColl, Alison McNeilly, Alyson A Miller, Scott Miners, Vincent Mok, Michael O’Sullivan, Bettina Platt, Emily S Sena, Matthew Sharp, Patrick Strangward, Stefan Szymkowiak, Rhian M Touyz, Rebecca C Trueman, Claire White, Chris McCabe, Lorraine M Work, Terence J Quinn

**Affiliations:** 1 Institute of Cardiovascular & Medical Sciences, College of Medical, Veterinary & Life Sciences, University of Glasgow, Glasgow, UK; 2 Centre for Discovery Brain Sciences, University of Edinburgh, Edinburgh, UK; 3 Lydia Becker Institute of Immunology and Inflammation, Division of Neuroscience and Experimental Psychology, School of Biological Sciences, Faculty of Biology, Medicine and Health, The University of Manchester, Manchester Academic Health Science Centre, Manchester, UK; 4 Stroke Trials Unit, Division of Clinical Neuroscience, University of Nottingham, Nottingham, UK; 5 Faculty of Medicine, University of Southampton, Southampton, UK; 6 University of Strathclyde, Strathclyde Institute of Pharmacy and Biomedical Science, Glasgow, UK; 7 The Department of Anatomy, Brain Health Research Centre and Brain Research New Zealand, University of Otago, Dunedin, New Zealand; 8 School of Life Sciences, University of Nottingham, Nottingham , UK; 9 School of Psychology, Cardiff University, Cardiff, UK; 10 Molecular & Clinical Sciences Research Institute, St George’s University of London, London, UK; 11 School of Psychology, University of Sussex, Brighton, UK; 12 Institute of Neuroscience, Newcastle University, Newcastle Upon Tyne, UK; 13 Institute of Clinical Neurosciences, University of Bristol, Bristol, UK; 14 Centre for Clinical Brain Sciences, University of Edinburgh, Edinburgh, UK; 15 UK Dementia Research Institute, Edinburgh Medical School, University of Edinburgh, Edinburgh, UK; 16 School of Medicine, University of Dundee, Ninewells Hospital, Dundee, Scotland; 17 Gerald Choa Neuroscience Centre, Therese Pei Fong Chow Research Centre for Prevention of Dementia, Division of Neurology, Department of Medicine and Therapeutics, The Chinese University of Hong Kong, Hong Kong; 18 Faculty of Medicine, The University of Queensland, Queensland, Australia; 19 Institute of Medical Sciences, University of Aberdeen, Aberdeen, Scotland; 20 Institute of Neuroscience & Psychology, College of Medical, Veterinary & Life Sciences, University of Glasgow, Glasgow, UK

**Keywords:** Dementia, methodology, outcomes, questionnaire, vascular cognitive impairment

## Abstract

Assessment of outcome in preclinical studies of vascular cognitive impairment (VCI) is heterogenous. Through an ARUK Scottish Network supported questionnaire and workshop (mostly UK-based researchers), we aimed to determine underlying variability and what could be implemented to overcome identified challenges. Twelve UK VCI research centres were identified and invited to complete a questionnaire and attend a one-day workshop. Questionnaire responses demonstrated agreement that outcome assessments in VCI preclinical research vary by group and even those common across groups, may be performed differently. From the workshop, six themes were discussed: issues with preclinical models, reasons for choosing functional assessments, issues in interpretation of functional assessments, describing and reporting functional outcome assessments, sharing resources and expertise, and standardization of outcomes. Eight consensus points emerged demonstrating broadly that the chosen assessment should reflect the deficit being measured, and therefore that one assessment does not suit all models; guidance/standardisation on recording VCI outcome reporting is needed and that uniformity would be aided by a platform to share expertise, material, protocols and procedures thus reducing heterogeneity and so increasing potential for collaboration, comparison and replication. As a result of the workshop, UK wide consensus statements were agreed and future priorities for preclinical research identified.

## Introduction

Dementia is a clinical and research priority condition,^1^ yet despite our best efforts we still have few proven treatments for the disease.^2,3^ In vascular-related dementias, the therapeutic options are especially limited.^4^ Recent years have seen concerted efforts to try and progress the dementia research agenda and improve the dementia drug discovery pipeline.^5^ As in many disease areas, with stroke being the most relevant example, a rate limiting step in translational dementia research has been inconsistent or inefficient study outcomes measurements.^6^

Reviews of the literature suggest that heterogeneity in outcome assessment is a particular issue across all dementia studies.^7^ Our objectives were to describe how outcomes are assessed in pre-clinical vascular cognition studies and to explore expert opinions on the optimal approaches to testing. We describe the UK perspective, through a focussed review of the literature, convening a one-day workshop, complemented by questionnaire data from those centres with active pre-clinical vascular dementia research programs. In this report, we provide context by describing the issues and potential solutions to outcome assessment in other disease areas, notably stroke. The findings of a literature review and the questionnaire and opinions from the workshop are then analysed and summarised into a set of consensus statements and priority directions for future research.

### Vascular cognitive impairment research

Vascular cognitive impairment (VCI) is a term that encompasses a spectrum of cognitive syndromes.^8^ The common feature is that the predominant underlying pathology is of vascular origin. VCI includes all forms of vascular dementia, cerebral amyloid angiopathy and also milder cognitive phenotypes that do not meet the dementia criteria. Vascular disease is generally said to be the second most common cause of dementia after Alzheimer’s disease (AD).^8^ However, there is increasing evidence that dementia in older age is driven by more than one process and a vascular component mixed with AD or other pathologies is common.^9^ Thus, within the VCI remit there is a spectrum of both cognitive syndromes and underlying pathological states. The two do not necessarily map neatly on to each other. Regardless of how VCI is categorised, it represents a major and increasing cause of disability.

Despite the importance of VCI, compared to other dementias such as AD, vascular causes of cognitive decline are relatively under-researched. The landscape is evolving and clinical, research and policy interest in VCI is increasing. The use of multi-centre cohorts,^10^ ‘big data’ and novel approaches to trials^11^ are all welcome developments but there remains an important role for pre-clinical research,^10^ not least because at present we have no proven and internationally licensed treatment options for VCI.

The traditional process of drug discovery and development has had only limited success in VCI and dementia in general. Many potential therapeutic agents have shown promise in pre-clinical studies and early human phase II studies; however, these were shown to have no beneficial effect when tested in definitive phase III randomised controlled trials (RCTs).^12^ Recent neutral results across a portfolio of putative AD treatments provide further evidence of the difficulty in the bench to bedside translation.^13^ One conclusion, which parts of the pharmaceutical industry are considering, is that this whole approach is misconceived. Before reaching such a conclusion, however, it should be asked whether there are key aspects that currently hamper translation that could be improved. Choice and translatability of outcome measures are one possible area. Certainly, for the research community, the message seems to be that the status quo is not working and that all aspects of the pipeline should be reconsidered. Choice of outcome assessment and the methods used to perform these assessments is an important aspect of study methodology and there may be potential to improve practice. Moving forward, a bedside to bench approach that better models the clinical condition is likely to uncover mechanisms that can be targeted pharmacologically or remedially for improved translation.

### Learning from stroke research

The themes described in our discussion of VCI research, namely: a substantial clinical problem with limited pharmacological treatments, multiple positive preclinical treatments and frequently disappointing phase III results, were all true of stroke research in the 1990s and early 2000s. Reflecting on the developments in contemporary stroke research can offer some guidance for how we approach VCI research. A series of high profile neutral results for neuro-protectant agents was a driver for the stroke research community to critically reflect on their approach to drug development.^14^ Various working groups were convened, that incorporated stakeholders from all aspects of the translational pathway including industry and regulatory representatives. Documents and guidance, such as the materials produced by the various Stroke Treatment Academic Industry Roundtable (STAIR) meetings and the Collaborative Approach to Meta-Analysis and Review of Animal Data from Experimental Studies (CAMARADES) group, have helped increase efficiency and rigor in many aspects of the translational approach.^15–17^ Many of the recommendations of STAIR and CAMARADES have been adopted by funders, journal editors and regulatory agencies. It is difficult to quantify the success of these programs and we should be cautious in ascribing changes in stroke research success to these initiatives. However, it is encouraging to see the growth in positive trial results from new interventional, pharmaceutical and device-based interventions in stroke.^18–20^

A key consideration of STAIR was to improve methods and reporting of outcomes and trial end-points. It was recognised that stroke trialists were using a variety of different methods to quantify treatment effects with no consensus on optimal measures or approaches.^21^ This heterogeneity in assessment is problematic as it complicates any attempt to compare results or pool data for meta-analyses. Heterogeneity in outcome assessment is particularly apparent when cognitive tests are used as outcomes in stroke studies.^22,23^ In fact, a recent review suggested that there were more assessments than there were trials. In response, a set of core outcomes measures for stroke trials were suggested with preferred tests for describing stroke impairments and activity limitations.^24^ The concept of core outcomes for stroke trials has been adopted by many specialist societies and by regulatory agencies.^25,26^ The move towards a more standardised approach to outcome assessment is not unique to stroke. In many other disease areas, heterogeneity and inconsistency in outcome assessment are noted. The Core Outcome Measures in Effectiveness Trials (COMET) initiative aims to create and apply core outcome sets.^27^ COMET guidance has created core outcome sets for many disease areas, recognising that defining a preferred test should not restrict researchers from measuring other study specific endpoints of their choice.^28^

A recurring theme from the work around stroke outcomes was that a stroke trial should have some measure of functional recovery. For many stroke trials, the modified Rankin Scale (mRS) was the primary outcome of choice.^29^ Although widely used, mRS was not designed for use as trial outcome measure and there were certain problems in the psychometric properties of the scale, most notably around inter-observer variation in the grade assigned.^30^ Having settled on a preferred outcome measure, the stroke research community developed methods to standardise mRS, including training,^31^ structured approaches to questioning,^32^ expert adjudication panels^33^ and novel methods for analysis.^34,35^ In respect of measuring cognitive outcomes in acute stroke trials, few did (e.g. see ENOS Trial Investigators^36^) in spite of recommendations to do so.^22,23^ The substantial progress made around outcome assessment in clinical stroke research should serve as an exemplar for developing assessment strategies in other research fields. Stroke assessment and VCI assessment are far from synonymous, but there is sufficient commonality in the diseases, the models and the research challenges for the VCI pre-clinical research to explore the potential application of recent methods used to raise standards in stroke research outcome assessment.

### Review of the literature

To inform and give context to this work, we systematically reviewed the literature on outcome assessment in pre-clinical VCI research. This work was taken from a larger body of work with a more general pre-clinical, functional outcomes remit.^37^ To complement this search of electronic literature databases, a search of the COMET and EQUATOR (Enhancing the Quality and Transparency Of health Research) databases found no existing work on core outcome sets for VCI, although preferred outcomes for clinical VCI research are in preparation. In addition, we consulted the consensus working group with the Stroke Recovery Research Round Table (SRRRII) proceedings of a consensus working groups that is focusing on stroke-induced cognitive impairment.^38^

We assessed outcomes used in selected, published pre-clinical VCI studies over a decade’s worth of research (2005–2015) with dates chosen to reflect a period of growth in the VCI research space. This was part of a larger project analysing preclinical stroke research that has been published and full details of methods and results are available in the parent publication.^37^ From this work, we extracted data specific to VCI research.

**Figure 1.**
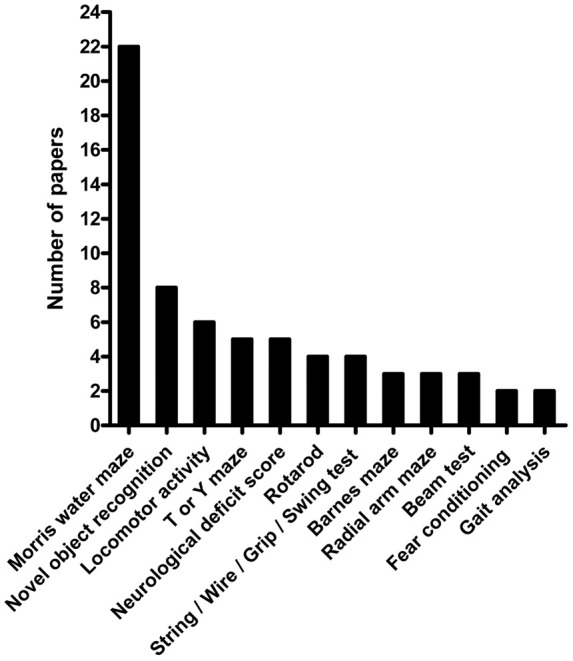
Outcome assessments used in across a decade of published VCI pre-clinical research. Outcomes described across VCI research published (*n* = 37 articles) in selected journals 2005–2015 in more than two articles.

Across a decade of research, we found that VCI was less frequently studied than other aspects of stroke. In those papers with a predominant VCI focus, there was substantial heterogeneity in outcome assessment with many tests used in two papers or more (Figure 1). In addition to those listed, the following outcome assessments were described in only one paper: adhesive removal test, buried food retrieval (olfactory), circular hole board (dry maze), elevated plus maze, inclined plane test, limb placement test, nest construction test, porsolt swim test, social interaction in novel environment. For the assessment that was used most often, the Morris Water Maze (MWM), we found marked inconsistency in how the test was performed and reported. We had hoped to categorise the tests according to the cognitive domain being tested, but such data were infrequently reported. These VCI findings mirror the results of a systematic analysis of studies using AD mouse models.^39^ In transgenic mouse studies, there was heterogeneity in assessments employed, with the MWM was being the most commonly used test, yet marked variations in the conduct and analysis of the MWM testing were noted. There was a clear sex bias with 62% of studies using only male animals, 3% only female, 16% both sexes and 19% did not specify sex used.^37^ Whilst we acknowledge a clear bias towards using males in preclinical research exists, we support and encourage the need to undertake more research using both sexes (males and females) given known sex differences exist.

These data come with certain caveats, the search was restricted to priority journals with a cerebrovascular focus and so is not a comprehensive synthesis of VCI research. We also recognise that VCI research, models and outcomes continue to evolve and some of the newer models of VCI, for example those that model amyloid angiopathy, may be under-represented. Thus, the focus and time frame may omit other VCI models (e.g. hypoperfusion, small vessel disease relevant) which are more representative of the milder end of the VCI spectrum and for which arguably there has been more extensive behavioural testing. Accepting these limitations, our illustrative review offers some useful insights into outcome assessment in pre-clinical VCI work.

## Methods

We collated opinions and practices around pre-clinical VCI assessment in UK academic centres. With support from Alzheimer’s Research UK Scottish Network, we held a one-day workshop, complemented by a questionnaire of usual practice. The work had ethical approval from University of Glasgow, MVLS ethical committee (ref:200170103). This work is the first in a planned series designed to raise standards in pre-clinical VCI research.

### Sampling frame

Our approach was purposive, using our own knowledge of VCI research and also consulting with Alzheimer’s Research UK and Dementia Platform’s UK Vascular Experimental Medicine Group to identify UK academic centres with an active VCI research laboratory. From an initial approach, we asked respondents to name other researchers working in this space to allow for a snowball distribution. Our focus was on neurocognitive and behavioural outcomes. These are functional tests and do not include measures of pathology, neuroimaging or biomarkers. To align with the clinical research literature, we use the term ‘functional assessment’ to describe these tests.

### Questionnaire

We followed best practice in design, conduct and reporting of our questionnaire study.^40^ Academic centres that were involved in the workshop were first sent a short questionnaire by the study Principal Investigators (LW and TQ). The bespoke questionnaire was designed and piloted in-house before dissemination. The questionnaire focussed on the functional outcome assessments used in pre-clinical VCI research and large vessel stroke models by the respondents. Additional questions explored perceived heterogeneity in choice and application of functional outcome assessment measures. The questionnaire was sent electronically with an explanatory cover letter (or by post on request) and up to two reminder messages were sent if no response was received. No incentive was offered for completion. It was stated that completion of the questionnaire implied consent to participate and a separate consent form was not used. We aimed to include all relevant centres and so we did not perform sample size calculations. Questionnaires were collated, results entered into a database and described in aggregate using simple descriptive statistics. The questionnaire had space for free text comments, where these were completed verbatim comments were added to the results of the focus group discussion described below. Recognising that more than one PI may work in a centre, we accepted more than one response from each Institute but asked that each team should submit only one questionnaire. The full questionnaire is available in the Supplementary Materials (Figure S1).

### Workshop

We convened a one-day workshop on June 11 2018 in the Institute of Cardiovascular and Medical Sciences, University of Glasgow, UK. We used the same distribution list as the questionnaire. Primary invitation was sent to senior researchers within a centre, who also nominated early career researchers to participate where appropriate. We used a focus group approach, with a moderator facilitating a semi-structured discussion with a small group, resulting in three groups with eight participants, one facilitator and one scribe. The facilitator was one of three senior researchers with an interest in VCI (LW, TQ, CMcC), and the scribe was an early career researcher who took notes during the discussions.

The day began with an overview presentation that included the results of the literature review (described above) and preliminary results from the questionnaire. Two 1-h discussions were then held, the first focussing on assessments used in the participants’ laboratory and the second focussing on the application of tests for VCI and other relevant models. Topics covered during the discussions were guided by the group with the facilitator ensuring that the following themes were all addressed: choice of tests, rationale for choice, application of tests, core outcomes sets. Each group offered feedback on their discussions with the others in the room and this conversation was also recorded by the scribe.

Responses were recorded as free text and these data were shared with one of the research team (TQ) who collated the comments and categorised as common themes emerged. This process was continued until all free text comments were categorised. The resulting summary was electronically shared with all participants for comment or correction. The statements presented represent a final consensus on the topic. We provide a narrative summary of the discussion, but as per our ethical approvals, we do not attribute responses to a particular participant.

## Results

### Questionnaire about choice of VCI outcome measures

We identified 12 centres in the UK with an active VCI research portfolio. For the questionnaire, we received replies from 23 researchers, with responses from 11/12 of the centres approached. The majority, 18/23 (78%) of respondents, said they did not agree that research groups used the same tests in VCI research. Similarly, the majority of responses, 21/23 (91%), said that the approaches used and scoring were not consistent.

Respondents were asked to list all outcome measures that they used in VCI studies (Question 2, Supplementary Figure 1), which generated a list of 23 assessments, most of which were used by a single respondent (Table 1). We used the test names as returned by the respondents and did not try to categorise these. It could be argued that some responses with different names are essentially the same test. Without knowing the detail of the test, we opted not to combine similar responses and instead present the responses as submitted. Respondents interpreted the ‘functional’ assessment rubric in different ways and some of the tests reported would not classically be considered as neurobehavioral assessments. The most commonly used test was Novel Object Recognition followed by the MWM. The original intention was to group test by cognitive domain assessed. This was not possible as some of the tests mapped across to various domains. This difficulty in categorising VCI outcome assessments was a discussion point during our workshop.

**Table 1.** Reported outcome measures used in UK VCI pre-clinical research centres.

Test	No	Test	No	Test	No
Novel object recognition	5	Classical conditioning	1	Pole test	1
Morris water maze	4	Forced swimming task	1	Rotarod	1
Radial arm maze	3	Locomotor activity	1	Spatial recognition	1
Barnes maze	2	Modified Bederson	1	Sticky label	1
Neurovascular coupling	2	Nest building	1	T maze	1
Operant conditioning	2	Neuronal activity	1	Tail suspension table	1
Y maze	2	Open field	1	Touchscreen tests	1
Asymmetry	1	Paw placement	1	Visual acuity	1

### Workshop discussion summaries

The workshop included 30 participants, representing 12 different UK centres. Participants were 14 senior researchers (head of department, chair or principal investigator of a research group) and 16 early career researchers. The comments generated were collated into six themes, each described below. In addition, the group agreed on a set of consensus statements around current practice in pre-clinical VCI functional outcomes research and some priorities for future work in this area. The themes, consensus statements and research priorities were shared with all contributors and other international experts. Revisions were collated centrally by the lead authors (LMW, TJQ), incorporated without attribution and revised materials were shared with the group. Process continued until no more changes were suggested.

#### Theme 1: Issues with pre-clinical VCI models

The models most commonly used by the participants’ research groups were vessel occlusion models (permanent or transient), microembolic models, models using vascular risk factors and genetic vasculopathy models. This is not an exhaustive list of models used by the participants and many other models were all discussed. Each group raised issues around the suitability of functional assessment in some of the commonly used VCI models. Many groups use murine models but there was debate around how well these models can capture the cognitive phenotypes similar to those seen in clinical VCI.^41^ There may be little benefit in designing a test strategy to capture issues such as dysexecutive problems or language impairments, if the animal being tested does not have a basic level of ability in these domains even in health. Some participants felt that aspects of executive function could be assessed in mice, using attentional control, working memory, rule learning and reversal-based assessment. Some other laboratories are developing models in other larger species such as pig.^42^ The advantages to this approach were recognised, but these had to be weighed against the increased costs and ethical considerations of working with these animals. Whilst larger animals including non-human primates show some advantages for assessing cognitive impairments over rodents, there still remains the issue of not being able to assess language and speech.

Another aspect of VCI models that was discussed was the role of lesion specific modelling versus models that create a more diffuse brain insult. It was agreed that these different models require different approaches to functional assessment – domain specific testing may be relevant to models with precise lesioning (e.g. focal endothelin-1 injection), while a more global testing strategy may be useful in diffuse injury (e.g. global hypoperfusion through bilateral carotid stenosis). Once again it was recognised that our limited understanding of the pathology of human VCI precluded any definitive statement on the optimal pre-clinical model that should be used. The agreement was that whichever model is employed, the outcome assessments must be relevant to the intended brain lesion. An alternative argument was made, rather than have the model dictate the outcome assessments, we could consider having the cognitive deficit of interest (i.e. the outcome) inform the choice of model.

#### Theme 2: Reasons for choosing specific functional assessments

All groups discussed the assessments that they used in their laboratory and the reasons why one test was preferred over another. A theme emerged that the rationale for using a particular assessment was often driven by practical considerations as well as science. Groups described the time and training investment required to develop a functional assessment protocol that is reproducible and robust for a particular VCI model. Once a protocol is established, this often meant that the included test was used in subsequent experiments and grant applications. Groups were resistant to changing established practice unless clear benefit could be demonstrated. It was recognised that the time and effort involved in becoming familiar with testing were a barrier to new groups beginning VCI research. All these points supported the calls for greater sharing of resource and expertise.

Some of the testing paradigms favoured in the participants’ centres had been modified from existing protocols used in non VCI-based models particularly AD and stroke. Ultimately it was recognised that choice of functional outcome assessment was not always driven by neuropsychological considerations. The example of the popular MWM was used, a test prevalent in VCI research but that does not capture frontal lobe anomalies involving executive dysfunction, attention, processing speed, reaction time and object recognition. Vascular cognitive impairments span multiple domains and therefore no single measure is going to be appropriate to all VCI research. Tests need to be validated for the domain of interest and if the function assessed spans more than one neuroanatomical hub, then more than one test may be required.

Some groups discussed the potential for new technologies, with translational relevance, to improve outcome assessment.^43^ The example most widely mentioned in resulting discussions was the use of touchscreen-based test batteries designed for rodent models but additionally relevant to the clinical setting. Touchscreen-based assessments have the potential to model human assessment tools and so could allow for greater pre-clinical and clinical harmonisation of assessment. Although groups were aware of digital platforms that had been used for testing, only one of the participants had expertise in using the technologies. The group also had concerns over whether the cost, and often lengthy protocol, justified adopting these techniques in preference to more established assessment strategies. There were also concerns over whether grant funding bodies would view these technologies as good value for money.

##### Theme 2: Consensus point

- The functional outcomes measures that are best suited to the study of VCI will often differ from measures used in Alzheimer’s or other neurodegenerative pre-clinical research.

#### Theme 3: Issues in the interpretation of functional assessments

There was a general recognition that all of the commonly used functional assessments had inherent limitations. It was felt that this did not invalidate the use of the tests but interpretation of the results came with certain caveats. The models used to induce VCI may not cause exclusive cognitive deficits. There is the potential for performance on functional tests to be confounded by other deficits for example a stroke motor deficit could bias timed cognitive tasks such as mazes.^44^ The same problem is seen when testing cognition in clinical stroke^45^ and only recently test batteries have been developed that take account of non-cognitive stroke deficits.^46^ In most centres, researchers screen animals before testing to ensure they are fit for the assessment. Assessment varied according to the model and the outcome being assessed and could include screening for motor, visual or other sensory impairments. It was felt that this could be formalised into a set of inclusion and exclusion criteria that must be met before testing and reported in results, a situation analogous to cognitive testing in clinical research studies.

Another potential confounder, albeit more difficult to quantify, is seen in the emotional and stress response associated with the testing paradigm itself. Many cognitive tests expose the animal to a novel situation or involve fear, reward, food deprivation, etc. These factors may bias test performance, although it was felt that this was less of an issue if a control group is exposed to the same tests. Habituation of animals to behavioural tasks needs also to be considered and standardised. It was felt that experience and training could reduce but not entirely remove these confounding issues.

All of these issues lead to an inherent variability in test performance and this in turn may necessitate large numbers to demonstrate between group differences. Statistical power analysis should be required to define group sizes in VCI studies with functional outcomes to avoid underpowered studies. This was an argument to support standardisation of assessment and a move towards larger, multi-centre collaborative projects.

The functional tests discussed were all designed to assess issues at the level of impairment. In clinical research, outcomes may assess more complex constructs such as disability (activity) or handicap (participation).^47^ There was some discussion around whether higher level functional issues could be captured in pre-clinical models, for example through describing social behaviours, or daily activity such as nesting. It was recognised that animal housing and bedding would need to be standardised if these behaviours were to inform any outcome measure.

Some participants suggested that trying to make sense of functional assessments in pre-clinical models was, in fact, too ambitious. The question was raised as to whether assessment of cognitive impairment, with attendant need for equipment and experienced staff, was even necessary. Instead, some form of quantification of pathology, such as size of lesion (if appropriate to model) or change in a biomarker may be a sufficient outcome for preclinical research. However, it was also noted that in clinical research, relying solely on a surrogate outcome has given misleading results and as a result for stroke research, functional outcomes are now preferred.

Cognition is a complex construct with differing brain regions said to contribute to differing domains of cognitive performance, for example the most recent clinical definitions of neurocognitive disorder recognise attention, learning and memory, language, visuospatial, executive function and social cognition.^48^ For some of the more popular functional tests, such as the MWM, it was noted that it can be difficult to categorise exactly which cognitive domains are being assessed by the test of interest. The MWM is usually described in terms of learning and/or memory but other cognitive domains (e.g. visuospatial) are also involved in the execution of the task. Mapping the cognitive domains being assessed to the available tests could assist in choice of test strategies for future research. There was a recognition that working with behavioural neuroscientists and neuropsychologists can aid the design, conduct and interpretation of cognitive assessments, but that researchers with these skills were limited in preclinical academic centres.

##### Theme 3: Consensus points

- Choice of functional outcome in pre-clinical VCI research should take account of anticipated cognitive deficit, disease process being modelled and potential confounders.
- Heterogeneity in choice, application and scoring of outcomes in pre-clinical VCI research limits the potential for comparison, collaboration and replication.
- A standardised set of screening assessments (e.g. motor, vision) could prevent confounding of cognitive outcomes. These may be specific to the chosen cognitive assessments.

#### Theme 4: Describing and reporting functional outcome assessments

Whether or not assessments are standardised to a consensus SOP, the testing strategy should be described in sufficient detail to allow for replication in another centre. The groups noted that reporting of functional outcome assessment methods was variable and there is potential to improve practice. The success of initiatives such as the Animal Research Reporting of In-vivo Experiments (ARRIVE) and other guidelines in raising standards around reporting of pre-clinical research was noted.^49,50^ However, the ARRIVE reporting checklist does not give specific guidance around reporting of outcome assessment methods, specifically how assessments were performed.^51,52^

The complexity of functional assessment and the many potential factors that could influence test performance were considered. As well as the potential variation in the actual test, factors specific to the animal (time since lesion, age, sex), factors specific to the environment (time of day, temperature, level of enrichment) and factors specific to the researcher (experience, contact time) could all modify test performance. All these variables, and potentially many others, should be recorded and it was agreed that some guidance on the key factors that need to be described in protocols and papers would be useful.

Not all animals complete a test as expected. For example, one laboratory gave the example of mice that often do not perform MWM appropriately and consistently swim around the edge of the water rather than engage in the task. These animals will not contribute any meaningful data to analyses and are excluded. Such exclusions should be fully transparent and included in any report, perhaps using a CONSORT flow diagram style figure.

In the past, a barrier to the comprehensive descriptions of different methods had been imposed by the word limits imposed in journals. In present times, with greater availability and use of online supplementary materials, this was felt no longer to be an issue. Some delegates suggested that we could move towards greater use of video to document process and application of testing. It was noted that many journals now mandate the use of reporting guidance (if available) before a study is considered for publication.

##### Theme 4: Consensus point

- Standardisation and guidance on the reporting of outcomes in VCI research are needed.

#### Theme 5: Sharing resources and expertise

A theme that dominated all the discussions was the need for greater collaboration and co-design among centres. It was recognised that having different centres developing and improving methods and protocols for the same assessment,but working in isolation, was time and cost inefficient. Many centres have developed their own materials such as training manuals, standardised operating procedures (SOPS) and ‘troubleshooting’ guides for outcome assessment but these are often only available for in-house use. Greater networking and collaboration between centres could facilitate information sharing. The Home Office licence approvals required for pre-clinical VCI work were recognised as another example of a potentially valuable resource that is required by all laboratories and that could be easily shared between centres. In doing so this could lead to greater standardisation across the UK in the conduct of VCI studies, making the licensing procedure more efficient and streamlined, hopefully resulting in more rapid approval of new licences and amendments. Informal communication between laboratories, on its own may not be sufficient to ensure visibility of the various resources available. A free access to repository for training materials, SOPS, etc. was a suggestion that was met with approval from all delegates. Some members noted that in clinical stroke, online training in outcome assessment with certification had become standard.^31^ Similar training could be developed for certain VCI outcomes. As well as the potential time and cost savings of sharing resources, a single point of access repository could also allow the research community to map which outcomes had sufficient training materials and guidance which outcomes needed such materials to be developed.

##### Theme 5: Consensus point

- The VCI research community needs a platform for sharing SOPS, training, equipment and archived tissue.

#### Theme 6: Standardisation of outcomes

Groups were encouraged to discuss standardisation in choice and application of functional outcomes. Opinions on the utility of standardisation differed. Many recognised the potential benefits but some group members also voiced reservations about an overly prescriptive approach. Many participants mentioned that training in the application and interpretation of behavioural testing is not uniform across centres. In particular, there will be differences between dedicated behavioural laboratories and more units with a more general portfolio.

All groups discussed the potential of creating a core set of preferred functional outcome assessments. While this was broadly endorsed, there were some reservations. Outcomes should not be mandated and researchers should still have the flexibility to use tests of their choosing. In many circumstances, particularly in early phase experiments, the outcomes of interest need to be chosen to align with the hypothesis being tested. The work of COMET emphasises that autonomy in choice of outcomes and use of a core outcomes set are not mutually exclusive.^27^ The ideal scenario would involve a battery of different tests, including certain core assessments and also incorporating other tests specific to the experiment.

There was general agreement amongst the delegates that a ‘one size fits all’ approach is not suited to exploratory pre-clinical research. Functional outcomes of interest in pre-clinical VCI research span a variety of neurocognitive and behavioural domains. Rather than a single preferred outcome, the group felt that a preferable approach was to create a menu of preferred tests, with tests suggested for all the various domains of interest.

It was indicated that a move towards a standardised test battery would be premature as we do not yet have valid models that capture VCI and our understanding of clinical vascular dementia syndromes is still evolving. The counter argument was that recommendations around tests could change in-line with scientific progress and it would be wrong to delay moves towards standardisation while important pre-clinical VCI research was already in progress.

At a practical level, there was recognition that a consensus approach to outcome assessment that was described in a widely adopted standard operating procedure (SOP) would help with grant applications, scientific protocols and applications towards approvals required for animal-based research. Many groups discussed the increasing interest in multi-centre, pre-clinical randomised controlled trials (pRCTs), with the Multicentre Preclinical Animal Research Team (Multi-PART) program of research cited as an exemplar of this approach.^53^ However, it was recognised that this interest was not yet matched by a substantial increase in pRCT activity. For true multi-centre work, an agreed and standardised approach to outcome testing would be essential, along with full support of grant awarding bodies to fund such work. At the moment, pRCT science is still at an early phase and it may be difficult to mandate standardisation, when the approach is still being refined.

##### Theme 6: Consensus points

- A single mandated outcome assessment would not be suitable for a complex construct such as VCI.
- A menu of preferred assessments for each cognitive domain could improve standardisation.

## Discussion

Using a multi-modal approach, we attempted to capture current practice and perceptions around functional testing in pre-clinical VCI research. In this report, we analyse and summarise the results of our literature search, questionnaire survey and focus group. Our review of selected, published VCI research suggested substantial heterogeneity in the choice and application of cognitive tests. Also, that the majority of VCI studies use male animals and fail to address the issue of sex bias in pre-clinical research highlighting the need to improve this important area. Researchers should be encouraged to investigate both males and females in VCI studies. Our questionnaires showed that inconsistency in assessment is recognised as an issue by the research community. To give these results context and to explore the issues with greater granularity, we ran focus group discussions. Although there were differing opinions around certain aspects of testing, there was substantial common ground and we were able to agree on consensus statements and priorities for future research (Textboxes 1 and 2).

To ensure that our process was robust, we engaged with various stakeholder groups to ensure that we achieved broad representation from the UK research community. All of the centres that we approached participated in either the questionnaire or focus groups. We ensured we had representation both from senior research leaders and from those earlier in their research career who may have more direct, day-to-day experience with performing assessments. We sense checked our results with international experts, who noted no issues with generalisability of the main recommendations, and used an iterative approach to refining our consensus statements and research priorities.

There are also limitations to our approach. It is possible that our sampling frame missed UK research groups with an active VCI interest. We plan future collaborative methodological work that builds on this project and any centres that wish to contribute in the future can contact us. For this phase of the work, our focus was the UK. The VCI research space is international and in particular we recognise the work in this area undertaken by National Institute of Health and National Institute of Neurological Disorders and Stroke raising similar issues within the AD and Related Dementias Summit 2019.^52^ Our international groups are also working towards consensus in VCI, such as Stroke Recovery and Rehabilitation Roundtable who are developing guidelines for improved translation of cognitive assessments after stroke that span both preclinical and clinical assessments.^54^ In the present work, we limited the geographical scope of this first project as we recognised that approvals and regulation of pre-clinical research can vary internationally. However, in many respects, the UK sample may not necessarily differ from international experience as we use the same variety of methods as are employed globally. Before recommendations can be made around VCI assessment, we would wish to repeat the exercise with a broader, international group. The synthesis of the focus group discussion was as objective as possible but there is always an element of interpretation. To ensure that what we report is an accurate representation, we shared all the data with participants, incorporating feedback and suggested changes until no more alterations were needed.

Our discussion and recommendations were developed exclusively from the results of our literature review, questionnaire and consensus meeting and, accordingly, focused on outcome measures rather than choice of model. From these sources, the predominant models and approaches were rodent, for example in our literature review only 2% of relevant VCI studies used non-rodent models. This finding is in keeping with other recent reviews of VCI models, where the non-rodent models were all larger species (e.g. ruminants).^42^ The potential differences between mouse and rat VCI models were not a major feature of our review or others^55^ suggesting that these differences are perceived as less important than other factors in the VCI community. General messages are applicable, for example ensuring validity of assessments across animals and the trade-off of cost/access versus similarity to the human clinical condition. The use of drosophila and zebrafish is gaining increasing traction in Alzheimer’s disease dementia where exciting results are being produced with these species.^56^ Our results would suggest that these models have less visibility in the VCI field. Hence, while this paper focuses on outcome measures, we appreciate the importance of having a valid model and plan further work in this area.

The consensus conclusions and research priorities that resulted from this project are presented in the text boxes. We believe these will prove useful to researchers but also to funders and journal editors. We were encouraged by the willingness of the pre-clinical VCI research community to work together on this project and believe it could serve as an exemplar for future methodological work. Other areas that could benefit from similar consensus and guidance include choice of VCI models and methods of analysis. In other areas of stroke and dementia research, collaboration, consensus and pooling of resources have driven forward the research agenda.^57^ Hopefully, our experience in bringing together UK pre-clinical VCI researchers is the beginning of many future collaborative activities.

Textbox 1. UK consensus on pre-clinical VCI functional outcomes

**Table:** 

• Choice of functional outcome should take account of anticipated cognitive deficit, disease process of interest and potential confounders.
• Heterogeneity in choice, application and scoring of outcomes limits potential for comparison, collaboration and replication.
• A standardised set of screening assessments (e.g. motor, vision) could prevent confounding of cognitive outcomes. These may be specific to the chosen cognitive assessments.
• The VCI research community needs a platform for sharing SOPS, training, equipment and archived tissue.
• The functional outcome measures that are best suited to the study of VCI will often differ from measures used in Alzheimer’s or other neurodegenerative pre-clinical research.
• A single mandated outcome assessment is unlikely be suitable for a complex construct such as VCI.
• A menu of preferred assessments for each cognitive domain will improve standardisation.
• Standardisation and guidance on the reporting of outcomes in VCI research are needed.

Textbox 2. Future research directions for pre-clinical VCI functional outcomes

**Table:** 

• Mapping available outcome measures to (clinical) cognitive domains.
• Work with funders and journals to developing reporting guidelines for outcomes.
• Need to explore role of new technologies.
• Explore developing multicentre approaches to VCI research.
• Broaden this work to gain an international consensus.

## Supplemental Material

JCB910552 Supplemental Material - Supplemental material for UK consensus on pre-clinical vascular cognitive impairment functional outcomes assessment: questionnaire and workshop proceedings Click here for additional data file. Supplemental material, JCB910552 Supplemental Material for UK consensus on pre-clinical vascular cognitive impairment functional outcomes assessment: questionnaire and workshop proceedings by Aisling McFall, Tuuli M Hietamies, Ashton Bernard, Margaux Aimable, Stuart M Allan, Philip M Bath, Gaia Brezzo, Roxana O Carare, Hilary V Carswell, Andrew N Clarkson, Gillian Currie, Tracy D Farr, Jill H Fowler, Mark Good, Atticus H Hainsworth, Catherine Hall, Karen Horsburgh, Rajesh Kalaria, Patrick Kehoe, Catherine Lawrence, Malcolm Macleod, Barry W McColl, Alison McNeilly, Alyson A Miller, Scott Miners, Vincent Mok, Michael O’Sullivan, Bettina Platt, Emily S Sena, Matthew Sharp, Patrick Strangward, Stefan Szymkowiak, Rhian M Touyz, Rebecca C Trueman, Claire White, Chris McCabe, Lorraine M Work and Terence J Quinn in Journal of Cerebral Blood Flow & Metabolism
