# Supplementary material for: UK consensus on pre-clinical vascular cognitive impairment functional outcomes assessment: Questionnaire and workshop proceedings
Source: J Cereb Blood Flow Metab. 2020 Mar 9;40(7):1402–14. doi: 10.1177/0271678X20910552 (PMC7307003; doi:10.1177/0271678X20910552)
Supplement: JCB910552 Supplemental Material - Supplemental material for UK consensus on pre-clinical vascular cognitive impairment functional outcomes assessment: questionnaire and workshop proceedings [file JCB910552_Supplemental_Material.pdf]

ONLINE SUPPLEMENT

UK CONSENSUS ON PRE-CLINICAL VASCULAR COGNITIVE IMPAIRMENT FUNCTIONAL OUTCOMES ASSESSMENT: QUESTIONNAIRE AND WORKSHOP PROCEEDINGS

Aisling McFall[1]; Tuuli M. Hietamies[1]; Ashton Bernard[1]; Margaux Aimable[2]; Stuart M. Allan[3]; Philip M. Bath[4]; Gaia Brezzo[2]; Roxana O. Carare[5]; Hilary V. Carswell[6]; Andrew N. Clarkson[7]; Gillian Currie[2]; Tracy D. Farr[8]; Jill Fowler[2]; Mark Good[9]; Atticus H. Hainsworth[10]; Catherine Hall[11]; Karen Horsburgh[2]; Rajesh Kalaria[12]; Patrick Kehoe[13]; Catherine Lawrence[3]; Malcolm Macleod[14]; Alison McNeilly[15]; Alyson A. Miller[1]; Scott Miners[13]; Vincent Mok[16]; Michael O'Sullivan[17], Bettina Platt[18]; Emily S. Sena[14]; Matthew Sharp[5]; Patrick Strangeward[3]; Stefan Szymkowiak[2]; Rhian M. Touyz[1]; Rebecca C. Trueman[8]; Claire White[3]; Chris McCabe[19]; Lorraine M. Work[1*]; Terence J. Quinn[1]

<u>Supplementary Results/Tables</u>

Supplementary Materials 1: Questionnaire sent to active VCI research centres in UK

UNIVERSITY of GLASGOW

Thanks for taking the time to complete this short survey. You can answer as many or as few of the questions as you wish.

We are interested in the functional and cognitive assessments that you use in your vascular cognitive impairment (VCI) pre-clinical (animal) models.
An example of a functional assessment would be the Bederson scale an example of a cognitive assessment would be a Water Maze test.
There is space at the end for any other thoughts or comments you may wish to share.

Q1a: There are lots of functional and cognitive outcome assessments available for use in VCI.
Do you think that different research groups generally use the same tests?

YES ☐ **NO** ☐

Q1b: There are lots of different ways to perform and score functional and cognitive outcome assessments in VCI.
For a given test, do you think that different groups generally use the same approach to testing and scoring?

YES ☐ **NO** ☐

Q2. Which functional and cognitive outcomes do you use in your pre-clinical VCI research?
Write names in the table (*you can add more rows if you need more space)*.

Q3. We are hoping to run a program of research looking at VCI outcomes assessment. **Would you be interested in helping with any of the following activities?**
(tick all that you are interested in)

Participating in focus groups (in person) ☐

Participating in focus groups (on line) ☐

Contributing to papers / publications ☐

If you are happy to be contacted about these activities, please give your details below. *Completing this section is not mandatory, if you wish to remain anonymous it would be useful to have the name of your Institution.*

Name	Institution	Email

Q4.Can you suggest other researchers who may be interested in helping with this work?
These could be from your own institution or from other centres.

Name	Institution	Email (if known)

Any other thoughts or comments?

Thanks for taking the time to complete this survey, your input is really appreciated.
Completed surveys should be sent to: Terry.Quinn@glasgow.ac.uk
